# The Role of Auxin in Cell Wall Expansion

**DOI:** 10.3390/ijms19040951

**Published:** 2018-03-22

**Authors:** Mateusz Majda, Stéphanie Robert

**Affiliations:** Umeå Plant Science Centre (UPSC), Department of Forest Genetics and Plant Physiology, Swedish University of Agricultural Sciences, 901 83 Umeå, Sweden; mateusz.majda@slu.se

**Keywords:** cell expansion, cell wall, acid growth, auxin

## Abstract

Plant cells are surrounded by cell walls, which are dynamic structures displaying a strictly regulated balance between rigidity and flexibility. Walls are fairly rigid to provide support and protection, but also extensible, to allow cell growth, which is triggered by a high intracellular turgor pressure. Wall properties regulate the differential growth of the cell, resulting in a diversity of cell sizes and shapes. The plant hormone auxin is well known to stimulate cell elongation via increasing wall extensibility. Auxin participates in the regulation of cell wall properties by inducing wall loosening. Here, we review what is known on cell wall property regulation by auxin. We focus particularly on the auxin role during cell expansion linked directly to cell wall modifications. We also analyze downstream targets of transcriptional auxin signaling, which are related to the cell wall and could be linked to acid growth and the action of wall-loosening proteins. All together, this update elucidates the connection between hormonal signaling and cell wall synthesis and deposition.

## 1. Introduction

Plant cells exhibit a great diversity in size and shape. Meristematic cells are usually isodiametric and then differentiate by developing distinct forms to acquire specific functions. This is easily noticeable in cells such as tip-growing root hairs or multi-lobed pavement cells. In contrast with animal cells, plant cells have the particularity of being tightly connected to each other by their surrounding walls located outside of the plasma membrane. Cell walls are dynamic structures that act as an exoskeleton by participating in the establishment and maintenance of cell shape and by protecting the cell content from biological, chemical and biophysical sources of aggression [1,2]. Large plants such as trees are able to resist external forces due to the strength given by their cell walls [1]. Moreover, cell walls play a significant role in processes such as cell adhesion, intercellular communication and water movement [1,3]. Plant cell walls are classified into two groups; primary and secondary walls. The latter are usually present in specialized, non-growing cells and are beyond the scope of this review [1,2,3,4].

Primary cell walls (around 100–1000 nm thick in young growing cells) are essentially made of glucan-based cellulose microfibrils (CMFs) embedded in a highly hydrated matrix composed of pectins, hemicelluloses, structural proteins and proteoglycans [1,2,3,5]. The cell wall has to be fairly rigid, to provide support and protection, but also extensible, to allow cell expansion, which is driven by a strong intracellular turgor pressure [6,7,8,9,10,11]. A strictly regulated balance between wall rigidity and flexibility is therefore imperative to regulate the differential growth that results in such a diversity of cell sizes and shapes [2,7,12,13]. The plant hormone auxin is identified as a stimulator of cell elongation, as it increases cell wall extensibility [14,15,16]. Specifically, auxin regulates cell wall properties by initiating wall loosening [17,18]. The close link between hormonal action and cell wall synthesis and deposition has been investigated for many years, but much of the details still need to be clarified. Here we summarize what is currently known about the regulation of cell wall properties and the role of auxin in this process.

## 2. Plant Cell Walls

Plant cell walls are highly heterogeneous and cell wall composites vary among different species and cell types. Walls are very dynamic and their composition changes even within the same cell over time [1,19,20,21,22,23,24]. Nonetheless, the key polysaccharides are usually present and their structure, biosynthesis and interaction are summarized in this chapter.

### 2.1. Cellulose Microfibrils (CMFs)

Cell wall consists of different polymers including CMFs, which are embedded in components such as non-cellulosic polysaccharides and structural proteins. CMFs are the largest cell wall polysaccharides, composed of (1,4)-β-d-glucan parallel arrays assembled into long cylinders [25,26]. Due to their stiff and load-bearing properties, CMFs are resistant to tensional forces [1,2,3]. CMFs determine the direction of cell expansion. Indeed, their deposition and alignment define cell growth anisotropy [2,27,28], as shown by the characterization of cellulose-deficient *Arabidopsis* mutants, in which cell elongation is drastically reduced [29]. Cellulose synthesis takes place beneath the cell wall at the plasma membrane via large rosette complexes made of CELLULOSE SYNTHASEs (CESAs), and certainly other components such as KORRIGAN1 (KOR1), the function of which remains elusive [25,26,30,31]. The CMF patterning of the wall is mediated via cortical microtubules (cMT) and CESAs at the plasma membrane, with the orientation of CMFs within the wall following the pattern given by the cMTs [28,32,33,34,35,36,37].

### 2.2. Hemicelluloses and Pectins

CMFs are embedded in a matrix of hemicelluloses and pectins composed of various carbohydrates that display complex glyosidic linkages. In dicotyledons such as *Arabidopsis*, pectins and hemicellulose xyloglucans (XyGs) are the most abundant cell wall components [1]. XyGs are found mainly in primary cell walls and are thought to participate in cell wall extension during cell elongation [38,39,40,41]. XyGs are composed of (1,4)-β-d-glucan chains, with side chains consisting of galactose, fucose and xylose residues [42]. XyGs influence wall extensibility and stiffness, as cell walls in an *Arabidopsis* double *xyloglucan xylosyltransferases* mutant (*xxt1 xxt2*) are softer and weaker than walls in the wild type [7,43]. Mannans and heteromannans are hemicelluloses that are abundant in mosses, lycophytes and in the secondary cell walls of gymnosperms [42,44]. Other hemicelluloses such as xylans, heteroxylans and (1,3;1,4)-β-d-glucans are highly represented in monocotyledons (cereals and grasses) and in secondary cell walls [1,5].

Pectins play an important role in the regulation of wall properties, because they control wall porosity and hydration, which causes wall swelling and influences wall thickness. Moreover, pectins adjust wall extensibility by influencing the alignment of CMFs and form the middle lamella, an adhesive compartment between two adjacent cell walls [45,46,47,48]. Pectins are composed of highly heterogeneous polysaccharides, among which four main elements can be distinguished: homogalacturonan (HG), rhamnogalacturonan I (RGI), rhamnogalacturonan II (RGII) and xylogalacturonan (XGA) [45,49,50,51,52]. HG often contains highly methylesterified galacturonic acid residues, while RGI is more complex and is composed of alternating galacturonic acid and rhamnose with galactose, arabinose or arabinogalactans forming side chains [45,47,49,53]. A common feature of RGII is the presence of borate esters between RGII-specific sugar residues [3,45,49,54].

Non-cellulosic cell wall components are synthesized in the Golgi apparatus, packed into vesicles and trafficked along actin filaments (AFs) to the wall [33,55,56,57,58]. The walls of actively growing cells display a porous structure, which allows polysaccharide to move relatively to each other (such as sliding) within the wall [7,59]. Polysaccharide synthesis is carried out by synthases, which catalyze the polymerization of sugar residues, and glycosyltransferases, which connect the monosaccharides and short oligosaccharides to the polymer chains [1,60].

### 2.3. Structural Proteins

Beside polysaccharides, the cell wall contains various structural (non-enzymatic) proteins, which regulate its formation and growth [2,61]. Among these structural proteins, EXPANSINs (EXPs), EXTENSINs (EXTs), and ARABINOGALACTAN PROTEINs (AGPs) are well-characterized as regulating wall expansion [62,63]. EXPs are defined as wall-loosening proteins, enhancing wall expansion in acidic pH [64], and will later be discussed in the context of auxin-mediated cell wall expansion. Other structural glycoproteins, EXTs, are required for cell wall assembly [65,66,67,68], as the EXT3-defective *Arabidopsis* mutant root-, shoot-, hypocotyl-defective *(rsh*) presents defective wall formation [2,69]. AGPs play a role in plant protection against pathogens, and additionally, an increased amount of AGPs can be observed in wounded plants [61,70]. AGPs are known to specifically control pollen tube growth [61], but are also thought to regulate overall plant development [71].

### 2.4. Interactions within the Cell Wall

Cell wall physical properties are maintained through the interactions among its polysaccharides [3,72]. A new model displaying the interactions between different cell wall polymers has been recently presented, in which “biochemical hotspots” crosslink different polysaccharides [7,73]. These hotspots are present between CMFs and XyGs, but also among different CMFs, connecting them to each other [7,73,74,75]. This interesting model updates the previous theory, which was based on the wall being composed of separated CMFs, which could be cross-linked to either XyGs, in order to reinforce the wall, and/or pectins, in order to soften the wall [5,76].

Crosslinking of CMFs with XyGs increases wall mechanical resistance [77,78,79,80,81,82]. XyGs are important for the separation of CMFs, as the XyG-deficient *xxt1 xxt2* mutant is characterized by tightly compact CMFs [7,43]. XyG-CMF interactions are modulated by XYLOGLUCAN ENDOTRANSGLUCOSYLASE/HYDROLASEs (XTHs), which either catalyze the linkage of the XyGs to cellulose (strengthening the wall) or hydrolyze the breaking of the link of XyGs with CMFs (loosening the wall) [83,84,85,86,87,88,89,90]. During cell development, pectins are regularly delivered and inserted into the wall matrix, which suggests that their presence and abundance might regulate wall extensibility. Pectins can either enhance wall expansion by promoting movement of the CMFs or maintain CMFs in non-growing cell wall zones [91,92,93,94,95,96]. Moreover, different pectin domains crosslink to each other via calcium and boron bonds [1,47,49]. These connections are modified by PECTIN METHYLESTERASEs (PMEs), which regulate the crosslinking of pectins to calcium ions. Methyl-esterification (addition of methyl groups) decreases the ability of HGs to form crosslinks with calcium ions, causing softening of the wall. Accordingly, de-methyl-esterification (removal of the methyl groups) increases HG capacity to crosslink to calcium ions, which causes wall stiffening, compaction and enhanced adhesion [97,98]. Intriguingly, auxin has been shown to reduce the stiffness of the cell wall through demethylesterification of pectins in the shoot apex leading to organ outgrowth [99]. On the other hand, RGII chains are connected to each other through borate diester bonds, influencing wall hydration and thickness [47]. Arabinans and arabinogalactans are known to induce cell wall swelling, decreasing its stiffness while increasing its extensibility [100,101]. In summary, the cell wall is composed of a range of different polysaccharides, whose abundance and interactions determine its properties and regulate cell growth.

## 3. The Role of Auxin in Wall Extension

Water accumulation in the vacuole induces high turgor pressure, which drives plant cell growth. This strong tensile stress presses against the plasma membrane, leading to the stretching of the cell wall polysaccharides. The wall needs to be moderately rigid to oppose this turgor pressure, to avoid breaking. However, the wall also has to adapt its composition by modifying and constantly adding polysaccharides to allow cell extension [7,59,102,103]. 

Cell wall expansion and overall cell growth is regulated via several factors, including plant hormones. Among them, auxin plays a vital role in controlling plant growth and development via promotion of cell division (proliferation), growth (expansion, elongation) and differentiation [15,16,104,105,106,107,108]. Enlargement of the cell occurs prior to cell division, however, no changes are observed in the vacuole size at this stage. On the other hand, cell expansion includes vacuole extension and is defined as a turgor-driven increase in cell size, which is controlled by the cell wall capacity to extend. Cell expansion is related to an increased ploidy level (endoreduplication), cellular vacuolization and differentiation [106,109]. Almost four decades ago, auxin or indole-3-acetic acid (IAA) was implicated for the first time in cell wall loosening and cell expansion via modifications of cell wall composition. IAA causes pectin polymerization, and increases pectin viscosity and XyG depolymerization [110].

In this second part, we discuss the auxin role during cell expansion and its direct link to the changes occurring in the cell wall [111]. Auxin activates the expression of cell wall-related genes and stimulates the synthesis of proton pumps, which leads to apoplast acidification [106]. Auxin also activates plasma membrane (PM) H^+^-ATPases through upregulating the phosphorylation of the penultimate of threonine of PM H^+^-ATPases, leading to apoplast acidification [112]. In an acidic environment, wall-loosening proteins are active and cause wall enlargement. The changes in the wall trigger the cell to activate calcium channels, which pump calcium into the wall and increase the pH, causing growth cessation. Finally, auxin acts on the cytoskeleton (AFs and cMTs) through RHO OF PLANTS (ROP) GUANOSINE-5′-TRIPHOSPHATASES (GTPases) and promotes trafficking of vesicles containing new cell wall material [113,114,115,116].

### 3.1. Auxin Signaling Stimulates Cell Elongation

*Arabidopsis* seedling hypocotyls elongate exclusively by cell expansion, making this organ a model system in which to investigate the contribution of auxin signaling to cell elongation [111,117]. Auxin acts through the TRANSPORT INHIBITOR RESISTANT 1/AUXIN SIGNALING F-BOX (TIR1/AFB) nuclear auxin receptor family, the degradation of the transcriptional regulators AUXIN/INDOLE-3-ACETIC ACID (AUX/IAAs) and the AUXIN RESPONSE FACTORs (ARFs), which mediate different transcriptional responses [117,118]. TIR1/AFBs are part of the Skp1/Cullin/F-box (SCF) complex, which promotes degradation of AUX/IAAs, which otherwise repress auxin-mediated transcription [119] through the interactions with ARFs in the absence of auxin. Once the concentration of auxin increases, the hormone mediates the linkage of TIR1/AFBs with AUX/IAAs and the degradation of the latter through proteasomal activity [120,121,122,123]. Different *Arabidopsis* AUX/IAA mutants such as *auxin resistant/indole-3-acetic acid inducible* (*axr2/iaa7*, *axr5/iaa1*, *axr3/iaa17*), or *short hypocotyl/indole-3-acetic acid inducible* (*shy2/iaa3*) display cell expansion defects [106,124,125], indicating that auxin induces cell expansion through the degradation of AUX/IAAs. ARFs are transcription factors that bind to the promoters of auxin-responsive genes [122,126,127,128]. Among the 22 ARFs in *Arabidopsis,* ARF7 has been shown to positively regulate the expression of *EXP8* [129], thus playing an essential role in extensive cell growth [130].

### 3.2. Auxin and Cell Wall-Related Genes

Several studies have shown that auxin treatment can specifically change the expression of different genes. In one such study, seedlings treated with exogenous IAA displayed over 790 differentially regulated genes, 55% of which were upregulated [131]. Of these, we have only selected the upregulated genes that were specifically related to cell walls and classified them into groups related to different cell wall components such as cellulose, XyGs, pectins, and structural proteins (EXPs), peroxidases, and components related to secondary cell walls (Table 1). Clearly, auxin treatment results in the upregulation of key genes related to cell wall components, as can be seen in the table. However, it is important to note that the reported genes are not necessarily specifically related to cell elongation (wall expansion) and could be linked to different auxin-driven processes such as cell division, growth or differentiation. 

Several auxin-responsive genes have been shown to be upregulated in elongating dark-grown hypocotyls [130]. Interestingly, cell wall-related genes were also found to be upregulated in this elongating organ, among them genes encoding wall-loosening EXPs [64,132], XTHs [86,133], AGPs [134,135] and related to pectin modification [130,136]. The use of etiolated hypocotyls suggested these genes as being specifically related to cell elongation.

The synthetic auxin picloram (4-amino-3,5,6-trichloropicolinic acid) induces hypocotyl elongation [137]. A transcriptional analysis of differentially regulated genes was performed in elongating light-grown hypocotyls upon treatment with the picloram [117], revealing that picloram and IAA signaling act through common downstream transcriptional targets, which are thought to stimulate cell elongation. However, picloram treatment revealed 79% novel differentially regulated genes, which were not differentially regulated in the seedlings treated with IAA, suggesting that they might be specific for elongating cells. Upon picloram treatment, changes in the expression of 1193 auxin-responsive genes (of which 62% were upregulated) preceded the hypocotyl elongation. Moreover, these genes were identified as downstream targets of picloram-stimulated transcriptional auxin signaling [117]. Study of the gene ontology related to auxin-responsive genes showed over-representation of genes related to hormone signaling, cell wall and cell expansion [117,138,139]. We have decided to focus on the genes upregulated by picloram treatment in the hypocotyl and selected those that were specifically related to cell walls (Table 2). Similarly, to IAA treatment of whole seedlings, picloram treatment of hypocotyls induced genes related to cell wall elements such as cellulose, pectins, EXPs, XTHs, and PEROXIDASEs. However, members of these different classes were more widely represented in the hypocotyls, suggesting their potential role in cell elongation and wall extension. Analysis also revealed many upregulated genes related to pectin metabolism as well as novel players related to hemicelluloses, AGPs and other structural proteins. In summary, among differentially regulated genes upon auxin treatment, many are related to post-synthetic cell wall modifications. This indicates that auxin regulates cell expansion via stimulating changes in cell wall properties. However, the auxin concentrations used in these studies is not physiologically relevant and the interpretation of the results should be cautious.

### 3.3. Auxin Induces Acid Growth

**Figure 1:**
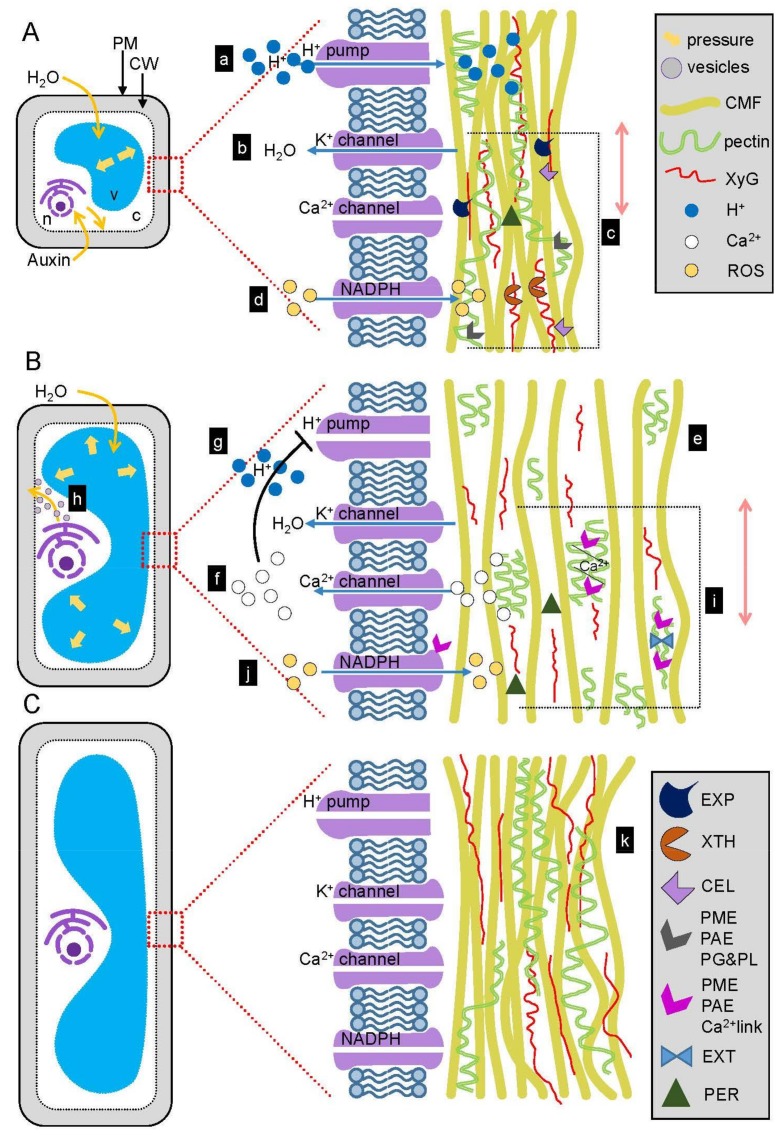
The role of auxin in cell wall expansion. Isodiametric plant cell preparing for elongation (**A**), undergoing elongation (**B**) and fully elongated (**C**). The cell contains intracellular structures such as nucleus (n) and vacuole(s) (v) in the cytosol (c) and is surrounded by plasma membrane (PM). Outside of the PM the cell wall (CW) is present (**A**–**C**). The PM consists of a phospholipid bilayer (in blue), while the cell wall consists of different polysaccharides such as cellulose microfibrils (CMFs in yellow), pectins (green double line), XyGs (red line) and other polysaccharides (not shown). Auxin activates plasma membrane H^+^-ATPase proton pumps, which pump protons (H^+^) into the wall matrix, leading to wall acidification (**a**). Acidification of the apoplast activates potassium channels, which transport potassium ions (K^+^) to the cytosol, stimulating water (H_2_O) uptake and maintaining tensile stress (yellow arrows in **A** and **B**) (**b**). Acidic pH activates wall-loosening proteins and enzymes, which loosen the connections between different cell wall polysaccharides (**c**). PMEs activate plasma membrane nicotinamide adenine dinucleotide phosphate (NADPH), transporting superoxide anions to the cell wall where they are converted to hydrogen peroxide (**d**). Wall-loosening proteins and enzymes cause CMF sliding and moving apart, which increases wall porosity (**e**). Cell wall extension leads to the activation of calcium channels and calcium efflux into the cytosol (**f**). Accumulation of cytosolic calcium inhibits H^+^-ATPase proton pumps and protoplast alkalization (**g**). Newly synthesized polysaccharides are inserted into the wall, where they arrive via vesicular trafficking (**h**). Wall alkalization activates PMEs, which in turn activate wall-degrading enzymes (**i**) and NADPH (**j**) causing crosslinking of the wall polysaccharides and growth cessation (**k**).

Auxin is known to induce acid growth (Figure 1), which is defined as the loosening of the walls at low pH, leading to an increase in wall extensibility and rapid cell elongation [14,16,140,141,142,143,144,145], through the TIR/AFB signaling machinery [146]. Auxin stimulates the activity of plasma membrane H^+^-ATPase proton pumps [147,148] (Figure 1(Aa)), which pump out protons (H^+^) to the wall matrix, leading to apoplast acidification (pH 4.5–6) [15,138,145,149]. This process induces the hyperpolarization of the plasma membrane and is regulated by the auxin-inducible SMALL AUXIN UP-RNA (SAUR) proteins [148]. Activation of potassium channels occurs and potassium ions are pumped into the cytosol (Figure 1(Ab)). The increasing concentration of potassium in the cytosol stimulates water uptake, which generates tensile stress, forcing the cell wall to extend [106,150,151]. Auxin not only stimulates the activity of proton pumps and potassium channels [150,151,152], but also induces the expression of genes encoding these proteins [150,151,152,153,154]. Note that auxin-sensitive proton pumps are mostly located in the epidermis [14,155], which is thought to be limiting for growth and is essential for shaping plant organs [154,155,156,157]. Moreover, different cells display distinct abilities to perceive acid growth; for instance, mature cells are less sensitive to acidic pH and extend less than young cells [158,159].

### 3.4. EXPANSINs Mediate Acid Growth

Auxin-induced acidic pH is required to activate EXPs (Figure 1(Ac)), which are specific, non-enzymatic wall-loosening proteins. EXPs were identified as inducing the relaxation of the walls in actively expanding hypocotyl cells of *Cucumis sativa* [64,160,161,162]. EXPs disintegrate polysaccharide networks by cutting and loosening connections between CMFs and non-cellulosic polysaccharides such as XyGs [161,163,164]. As a result, CMFs slide and move apart, promoting wall loosening, hydration and swelling. Interestingly, in plants exposed to gravitropic and light stimuli, EXP-encoding genes (*EXP1* and *EXP8*) are upregulated in elongating cells. This was observed before plant morphological changes appear, suggesting that auxin stimulates *EXP* expression, leading to the wall property changes [106,129].

### 3.5. Cellulose and Xyloglucan Modification during Wall Expansion

Auxin acidification induces cell wall modifications mediated by XTH and ENDO-(1,4)-β-d-GLUCANASEs (CELLULASEs), which loosen the connections between different cell wall polysaccharides within the wall matrix (Figure 1(Ac)) [3,165,166,167]. Auxin upregulates the expression of *XTH* family members (such as *XYLOGLUCAN ENDOTRANSGLUCOSYLASE*; *XET*) and *CELLULASEs* [106,117,168,169,170,171,172,173,174,175,176]. XTH proteins have been found in actively growing cells such as meristematic cells in the shoot apical meristem, leaf primordia and elongating roots, which are known to accumulate auxin [177]. In these cells, XTHs control cell expansion in two distinct ways. First, XTHs mediate the incorporation of nascent XyG chains into already existing XyGs, which suppress cell elongation [39]. Second, auxin-stimulated XTHs induce the modification of the polysaccharide network by cutting XyG backbones and by re-forming glycosidic linkages between different XyG chains within the already existing wall network. XTH-mediated cutting of XyGs provides short XyG fragments, which lead to loosening of the wall and promotion of wall rearrangement for cell elongation [2,39,178]. These short XyG fragments were also shown to interfere with auxin signaling, suggesting a negative feedback loop [179]. The degree of XyG fucosylation seems to also be important for regulation of cell wall expansion. Upon auxin treatment, non-elongating cells display enhanced abundance of fucosylated XyGs [180]. In the absence of exogenous auxin, cells containing fucosylated xyloglucan were shown to be elongating [39,179,181]. Interestingly, the auxin efflux-deficient *Arabidopsis* mutant *pin-formed1* (*pin1*) displays a progressive decrease of *XTH9* gene expression along its inflorescence stem (from the apex to the base) [182], which indicates that auxin might regulate the expression of the *XTH9* gene. However, auxin seems to have no influence on XTH action during root hair formation [183].

CELLULASEs hydrolyze glyosidic bonds in CMFs and are involved in cellulose formation/adjustment [173]. Auxin induces CELLULASE activity, leading to cleavage of load-bearing hemicellulose chains, which tether neighboring CMFs, and cleavage of cellulose chains. CELLULASEs modify the interactions between CMFs and XyGs, depolymerize XyG chains, producing short oligosaccharides [3,173], and promote wall loosening and extensibility [175]. In elongating stems of pea, auxin treatment induces the activity of CELLULASEs, which hydrolase the cellulose-XyG network, resulting in the release of wall-bound XyGs and their degradation [184].

### 3.6. Pectin Methylesterification and Its Consequences in Wall Loosening

Auxin induces low pH, which activates PECTIN METHYLESTERASE (PME) (Figure 1(Ac)) and inhibits PME INHIBITOR (PMEI). PMEs conduct random demethylesterification of initially homogenous HGs. Next, heterogenous HGs are deacetylated via PECTIN ACETYLESTERASEs (PAEs), neutralizing the galacturonyl residues, blocking their interactions with calcium ions. Finally, PECTATE LYASEs (PLs) [185] and POLYGALACTURONASEs (PGs) [186,187] depolymerize pectic chains, leading to loosened walls. As pectin depolymerizing enzymes, PLs and PGs provide short HG products called oligogalacturonides (OGAs) [98], which are small wall signaling molecules that act as potential ligands binding to WALL ASSOCIATED KINASE (WAK) membrane receptors [2,188,189,190]. OGA treatment inhibits pea stem elongation induced by auxin [191] and moreover, exogenously applied OGAs reduce auxin response and adventitious root formation promoted by auxin in *Arabidopsis* and tobacco, which indicates that OGAs might regulate auxin responses [190,192]. Accordingly, tobacco plants expressing fungal PG (depolymerizing HG and providing OGAs) display reduced sensitivity to auxin [193]. OGAs are also implicated in hydrogen peroxide (H_2_O_2_) production [194].

Hydrogen peroxide is a type of reactive oxygen species (ROS), which also includes superoxide anions (reactive oxygen anions) (O^−^_2_) and hydroxyl radicals (neutral form of the hydroxide ion OH^−^) (•OH), all of which are produced during plant metabolism, development and defense against pathogens. Although ROS cause cell damage and their levels must be strictly controlled by antioxidation, they also play a number of important roles such as in cell signaling and cell wall structure [195]. Auxin-induced PMEs activate the plasma membrane nicotinamide adenine dinucleotide phosphate (NADPH) OXIDASEs (Figure 1(Ad)), [2], which mediate transport of superoxide anions to the cell wall, where they are converted to hydrogen peroxide. PEROXIDASEs are enzymes abundant in the cell walls (Figure 1(Ac)), which use hydrogen peroxide and/or superoxide anions as substrates to catalyze a reaction producing hydroxyl radicals. These different ROS cause polymer breakdown, which leads to wall loosening during auxin-mediated cell extension [196,197,198]. Auxin has been proposed to stimulate the release of superoxide anions and hydroxyl radicals, leading to cell elongation [199]. Moreover, inducing the production of hydroxyl radicals causes an increase in wall extensibility, which indicates their role in inducing cell growth. On the other hand, the induction of superoxide anions causes the inhibition of auxin-induced growth [199].

The activity of wall-loosening proteins (EXPs) and enzymes such as XTHs, CELLULASEs and PMEs results in the sliding and moving apart of the CMFs (Figure 1(Be)) [2,64,160,200]. Loosening within the wall promotes its hydration and swelling. Next, wall porosity increases, creating a physical space for newly synthesized polysaccharides and proteins, which arrive via vesicule trafficking (Figure 1(Bh)). Nascent wall composites are secreted to the wall and integrated within the existing polysaccharide network thanks to modification of the polysaccharide interactions, through enzymatic hydrolysis, ligations and crosslinking. New polysaccharides must be added to compensate for wall stretching and thinning, in order to avoid the breaking of the wall. The cell wall surface area increases and the wall is irreversibly extended, which leads to wall relaxation and growth deceleration [59,102,103,201,202]. After the cell wall extends, information from the wall is transmitted back to the cytosol. Wall extension and relaxation stretch the plasma membrane and trigger calcium channel opening, leading to a calcium influx towards the cytosol (Figure 1(Bf)). The accumulation of cytosolic calcium inhibits the H^+^-ATPase proton pumps (Figure 1(Bg)) and stimulates a H^+^ influx towards the cell, causing apoplast alkalization [2,203,204,205].

### 3.7. Crosslinking of the Wall Polysaccharides

In an alkaline wall environment, PMEs conduct demethylesterification of HGs (Figure 1(Bi)). Next, PAEs deacetylate HGs making them accessible for calcium crosslinking, leading to pectin compaction [98]. PMEs also modify the methyl groups in HGs, which induces the cross-linking of polysaccharides and proteins (EXTs). This interaction causes wall dehydration and compaction, decreasing extensibility and growth [206,207,208,209,210,211,212,213,214]. Cell wall hydration is also regulated by enzymes such as the β-galactosidases (for example MUCILAGE-MODIFIED2 (MUM2) or SALT-OVERLY SENSITIVE5 (SOS5)), which are necessary for proper seed mucilage hydration. Mucilage in the *mum2 Arabidopsis* mutant contains an increased level of galactoses, which results in hydration defects [2,215,216,217]). ROS are also proposed to cross-link the wall polysaccharides or remove hydrogen atoms from polysaccharides, modifying the cell wall properties (Figure 1(Bj)). Together with PMEs, ROS promote wall dehydration and strengthening, which slows down growth (Figure 1(Ck)) [3,204,218,219,220,221]. However, Cosgrove (2005) [3] discusses the evidence that ROS play only a minor role in cell wall expansion, being responsible for only 1% of the extension. Growing cells produce very low amounts of ROS due to the fact that higher ROS concentrations can cause damage to the cells.

## 4. Conclusions

As the most external cell compartment, the cell wall is by necessity involved in plant cell growth. This has been demonstrated by analyzing different cell wall deficient mutants that display various growth defects. Indeed, the cell wall is a very dynamic cell composite, which is characterized by complex polysaccharide interactions and various modifications during cell development. Moreover, plant cells grow symplastically and they need to adjust their growth to the neighboring cells. Changes within the wall occur during turgor-driven cell growth, which is mediated via acidification of the wall and loosening of the connections between different cell wall polysaccharides. Acidic growth has been shown to be promoted by auxin, which activates structural proteins and enzymes such as EXPs, XTHs and PMEs, modifying the interactions between different cell wall polymers. Furthermore, progress in molecular biology has allowed us to connect auxin with the activation of the acidifying proteins (proton pumps, [221]) and numerous genes that are related to wall biosynthesis and modification. In summary, auxin plays a major role in regulating cell expansion through the activation of cell wall synthesis and modification-related genes. However, it still remains elusive as to how auxin regulates the modifications in the wall over time. Further development of in muro detection methods, which follow cell wall changes over cell development, will undoubtedly provide more clues about the temporal regulation of cell wall expansion and cell elongation by this master hormone.

## Figures and Tables

**Table 1:** Selected cell wall-related genes upregulated by IAA treatment in *Arabidopsis* seedlings (genes from [131]).

Cellulose Related	
*CELLULOSE SYNTHASE-LIKE*	*CSLC4; CSLC5*
**EXPANSIN related**	
*EXPANSIN*	*EXPA4; EXPA11*
*EXPANSIN-LIKE*	*EXLA3*
**XTH related**	
*XYLOGLUCAN ENDOTRANSGLUCOSYLASE/HYDROLASE*	*XTH18; XTH19; XTH23; XTH33*
*TOUCH*	*TCH2; TCH3; TCH4*
*XYLOSYLTRANSFERASE*	*XT1*
*ACT DOMAIN REPEAT 7*	*ACR7*
**Pectin related**	
*PECTIN METHYLESTERASE*	*PME1; PME34*
*PLANT INVERTASE/PECTIN METHYLESTERASE INHIBITOR SUPERFAMILY*	
*PECTIN ACETYLESTERASE*	*PAE11*
*GALACTURONOSYLTRANSFERASE-LIKE 10*	*GATL10*
*GALACTURONOSYLTRANSFERASE-LIKE*	*GATL3*
*GALACTAN SYNTHASE*	*GALS3*
*POLYGALACTURONASE INHIBITING PROTEIN 1*	*PGIP1*
**PEROXIDASE related**	
*PEROXIDASE SUPERFAMILY PROTEINS*	
**Secondary cell wall related**	
*OVATE FAMILY PROTEIN 1*	*OFP1*
*REDUCED IN LATERAL GROWTH 1*	*RUL1*
**Other/biosynthesis related**	
*EXORDIUM LIKE 2*	*EXL2*

**Table 2:** Selected cell wall-related genes upregulated by picloram treatment in elongating *Arabidopsis* hypocotyls (genes from [117]).

Cellulose Related	
*CELLULOSE SYNTHASE-LIKE*	*CSLC04; CSLC12; CSLD2; CSLD3*
*CELLULOSE SYNTHASE-INTERACTIVE PROTEIN 1*	*CSI1*
**EXPANSIN related**	
*EXPANSIN*	*EXPA1; XPA7; EXPA10; EXPA12; EXPA18*
*EXPANSIN-LIKE*	*EXLA1; EXLA2; EXLA3*
**XTH related**	
*XYLOGLUCAN ENDOTRANSGLUCOSYLASE/HYDROLASE*	*XTH8; XTH17; XTH18; XTH19 XTH23; XTH33*
*TOUCH 3*	*TCH3*
*XYLOSYLTRANSFERASE 1*	*XT1*
**Pectin related**	
*PECTIN METHYLESTERASE*	*PME2; PME41*
*PLANT INVERTASE/PECTIN METHYLESTERASE INHIBITOR SUPERFAMILY*	
*PECTIN METHYLESTERASE INIHIBITOR 7*	*PMEI7*
*PECTIN ACETYLESTERASE 11*	*PAE9; PAE11*
*PECTIN LYASE-LIKE SUPERFAMILY PROTEIN*	
*POLYGALACTURONASE INHIBITING PROTEIN 1*	*PGIP1; PGIP2*
*ARABINOXYLAN PECTIN ARABINOGALACTAN PROTEIN 1*	*APAP1*
*FRA8 HOMOLOG*	*F8H*
*GALACTAN SYNTHASE*	*GALS2; GALS3*
*BETA-GALACTOSIDASE*	*BGAL10; BGAL12*
*GALACTURONOSYLTRANSFERASE-LIKE*	*GATL7; GATL10*
*MALE GAMETOPHYTE DEFECTIVE 4*	*MGP4*
**Hemicellulose** **related**	
*ENDO-BETA-MANNASE 7*	*MAN7*
*ALPHA-XYLOSIDASE 1*	*XYL1*
*GLYCOSYLTRANSFERASE 18*	*GT18*
*MURUS 3*	*MUR3*
**AGP** **related**	
*ARABINOGALACTAN PROTEIN*	*AGP2; AGP9*
**PROLINE/LEUCIN-RICH PROTEIN related**	
*PROLINE-RICH PROTEIN 1*	*PRP1; PRP2*
*PROLINE-RICH PROTEIN-LIKE 1*	*PRPL1*
*LEUCINE-RICH REPEAT/EXTENSIN 2*	*LRX2*
*LEUCINE-RICH REPEAT PROTEIN*	*LRR1*
**Peroxidase related**	
*PEROXIDASE SUPERFAMILY PROTEIN*	
*PEROXIDASE 7*	*PER7; PRX25; PRX33*
**Secondary cell wall related**	
*PARVUS*	*PARVUS*
*TRANSPARENT TESTA 8*	*TT8*
*GLABRA 2*	*GL2*
**Signal Perception**	
*FORMIN HOMOLOGY*	*FH1; FH5*
*WALL ASSIOCIATED KINASE*	*WAK*
*THESEUS 1*	*THE1*
